# Identifying spin bias of nonsignificant findings in biomedical studies

**DOI:** 10.1186/s13104-023-06321-2

**Published:** 2023-05-03

**Authors:** Renée O’Leary, Giusy Rita Maria La Rosa, Robin Vernooij, Riccardo Polosa

**Affiliations:** 1 Center of Excellence for the Acceleration of Harm Reduction, University of Catania, Via Santa Sofia, 89 Torre Biologica 11 piano, 95123 Catania, Italy; 2 Department of Clinical and Experimental Medicine, University of Catania, Catania, Italy; 3 Department of Nephrology and Hypertension, University Medical Center Utrecht, Utrecht, The Netherlands; 4 Julius Center for Health Sciences and Primary Care, University Medical Center Utrecht, Utrecht University, Utrecht, The Netherlands

**Keywords:** Spin bias, Data discrepancies, Bias assessment, Peer review, Nonsignificant results, Systematic literature reviews, Quality assessment tools

## Abstract

**Objective:**

The purpose of this research note is to share a technique for the identification of spin bias that we developed as part of a living systematic review on the cardiovascular testing of e-cigarette substitution for cigarette smoking. While some researchers have remarked on the subjective nature of ascertaining spin bias, our technique objectively documents forms of spin bias arising from the misrepresentation of nonsignificant findings and from the omission of data.

**Results:**

We offer a two-step process for the identification of spin bias consisting of tracking data and findings and recording of data discrepancies by describing how the spin bias was produced in the text. In this research note, we give an example of the documentation of spin bias from our systematic review. Our experience was that nonsignificant results were presented as causal or even as significant in the Discussion of studies. Spin bias distorts scientific research and misleads readers; therefore it behooves peer reviewers and journal editors to make the effort to detect and correct it.

## Introduction

Our review team conducted an extensive quality assessment on the clinical studies we included in our living systematic review of the cardiovascular effects of e-cigarette substitution for cigarettes [1]. When we scrutinized the studies for many types of bias (drawn from the Oxford Centre for Evidence-Based Medicine *Catalogue of Bias* [2]), we were particularly concerned with spin bias. Spin bias is the distortion of data that misleads readers [3]. Spin bias is “a misrepresentation of study results, regardless of motive” [4]. One type of spin bias occurs when statistically nonsignificant results are reported as “showing an effect” [5] or where unjustified claims are made for results with *p-values* > 0.05 [6].

The reason for our vigilance for spin bias is because the use of e-cigarettes is a controversial and divisive field [7–9]. We, along with other researchers, are deeply concerned that “polarized stances on e-cigarettes will threaten the integrity of research” [9]. Calling out spin is critical because biased conclusions from studies garner media reporting [10] that becomes a source of misinformation, influencing public and clinicians’ opinions and actions. Senior members of the research team reported reading numerous instances of spin bias in e-cigarette studies. Would we find spin bias in the set of clinical studies included in our systematic review?

Yes, we did. Seven of 26 studies exhibited spin bias of nonsignificant findings (See Table 1). Spin bias may spring from “a strong position that relies more on their opinion than on the study results” [10], or it may be prompted as defense against publication bias against null results [11]. Whatever the motive, we developed a technique for an objective identification of spin bias for nonsignificant and misreported findings.

### Technique for the identification of spin bias

Our technique for identifying spin bias within an article has two steps.

First, the data and findings from the results, including in tables and figures and in appendices or supplementary materials, are tracked throughout their mentions in the study text. Data discrepancies and “pairs of statements that cannot both be true” [12] point to potential instances of spin bias. Our technique examines the misreporting or misrepresentation of nonsignificant data in the Discussion, but additionally this process can detect data discrepancies between the abstract and the conclusion of a study. Tracking can also reveal the omission of specific findings from the Discussion, another type of spin bias.

Second, the discrepancies identified in the data are reported with exact quotes (see Table 1) for objective identification. In our systematic review, many of the data discrepancies were between the data presented in a table or figure and what was stated in the text.

For non-significant findings in some instances, the spin is made with causal language, with a claim or by stating that an effect occurred where the finding was not significant [5, 10]. For example, in Table 1, there was no significant difference between e-cigarette and cigarette use on blood pressure, but in the Discussion the claim was that e-cigarettes had a lower impact than cigarettes. In some cases, the authors in their Discussion flatly stated that a finding was significant when it was reported as nonsignificant in the Results section. We observed this misreporting in two studies in our review.

For accuracy and completeness, two reviewers independently should check for data discrepancies and misreporting of nonsignificant findings. Differences in their assessments and observations should be resolved by discussion; this was our procedure. A third team member (in our review, the Project Leader) should verify all evidence of spin bias.

### Our systematic review: nonsignificant findings and spin bias in the studies

Table 1 displays the occurrences of spin bias we found in our systematic review with this technique and how we documented the evidence of spin bias.

**Table 1:** Data discrepancies and nonsignificant data spin

Data	Spin Bias: Section and Text	Study
Table 3 differences between TC and ENDS on blood pressure not significant	Discussion “blood pressure are less impacted by…[ENDS] than by TC suggests that they might be less detrimental”	[13]
Figure 7 no significant difference in PCO_2_ for ENDS with or without nicotine	Discussion “increased PCO_2_ after vaping without nicotine”	[14]
Table S3 LDL cholesterol changes not significant for ENDS or TC, not significant between study groups Results “Mean LDL cholesterol levels… according to the RMANCOVA analysis, they were not different between the two study groups”	Discussion “[ENDS] users also appeared to have greater decreases in…LDL cholesterol than [TC] smokers”	[15]
Results cSBP “no significant difference at any time point in the [ENDS] group”	Discussion “We demonstrate acute changes in central blood pressure for a short period of time after vaping a liquid with nicotine”	[16]
Results “lower systolic blood pressure among arms…was not statistically significant”	Results “greater reduction in systolic blood pressure for [ENDS] group than in the TC group”	[17]
Figure 3a differences between interventions are nonsignificant	Results “Statistically significant differences between interventions…Figs. 2b and 3a”	[18]
Table 4 no significant difference in HR minimum between control group and ENDS group.	Results “significant difference between C [control] and [ENDS]… for min[imum] HR”	[19]

Note: the studies used multiple terms for e-cigarettes and tobacco cigarettes. E-cigarettes terms are translated as ENDS (electronic nicotine delivery systems) and tobacco cigarettes as TC.

cSBP – central systolic blood pressure

ENDS – e-cigarettes

HR – heart rate

LDL – low-density lipoprotein

PCO_2_ – partial pressure of carbon dioxide

RMANCOVA – repeated measures analysis of covariance

TC – tobacco cigarette

## Critical discussion

It could be argued that authors could legitimately frame their nonsignificant findings by stating that “the findings *could be…*but not sufficient evidence.” This linguistic turn obscures the results of the actual data collected. The authors’ *could be* is likely their preferred hypothesis, yet nonsignificant data could be construed in any number of ways. The accurate statement would be that it was not a significant finding. Our living systematic review reported over 66% nonsignificant cardiovascular test results. This was important data indicating that e-cigarettes had no difference in cardiovascular effects than cigarettes. Nonsignificant findings provide evidence that should not be drowned out by the noise of speculations.

In the broader biomedical literature, misreporting and misinterpretation of study findings are evidently common practices that produce spin bias [10, 20, 21]. A comparison of 896 abstracts with their full text conclusions observed that 15–35% were “inconsistent” [22]. Another study documented that 22% of trials with nonsignificant results (75 of 346 studies) had high levels of spin bias in their Conclusions [5]. In our systematic review, 27% of the studies exhibited spin bias with nonsignificant results. Nonsignificant results appear to be the most prone to spin bias: “the only factor that seems consistently associated with spin is non-statistically significant results” [23, see also 5].

## Limitations

Certainly our technique for identifying spin bias requires further testing, evaluation, and validation. As far as we know, this is the only report on the identification of nonsignificant results and spin bias in the Discussion sections of published articles. Spin bias has been examined between an abstract and the text in randomized controlled trials [20, 22, 24, 25] and in systematic reviews [21, 26, 27]. Spin bias in abstracts is especially serious because many readers look only at the abstract.

Two recent checklists could incorporate our two-step process for identifying spin bias with nonsignificant findings. The recently published Quality Output Checklist and Content Assessment tool (QuOCCA) includes one item on the spin of nonsignificant results, but it purposefully excludes checking the Discussion because spin in that section of a study was “difficult to identify” [6]. This was not our experience. The Discussion section is a key section for reporting and interpreting results, and should be checked with the QuOCCA, not excluded. PRIOR (Preferred Reporting Items for Overviews of Reviews) [28] is another recently published tool with checks for reporting bias and data discrepancies. Both tools would be enriched with our technique to identify the spin of nonsignificant data, the most common reporting bias.

Our technique does not identify all instances of spin bias. It cannot document where particular datapoints, such as secondary outcomes or subgroup findings, have been over-emphasized over primary outcomes, although our technique does document findings which are omitted from the Discussion. Identifying spin bias from overgeneralizations and overstatements entails analyzing rhetoric and checking for ascertainment bias. Our technique will require adaptation to be useful for uncovering the spin bias from undocumented deviations from a clinical trial registry or protocol and the published study [29]. A rating for the intensity of spin bias could be based on the number of instances identified, with multiple occurrences a flag for potential researcher bias.

Finally, we can only wonder out loud about how much of an appetite there is among editors and peer reviewers to routinely take on an additional check. Spin bias distorts scientific research and misleads readers. Editors and peer reviewers should be vigilant for spin bias [5]. and “in theory, peer-reviewers and editors should determine whether the conclusions match the results” [10]. But it does not have to be all or nothing. Knowing that nonsignificant findings are the most common source of reporting bias, peer reviewers should spot check for how nonsignificant findings are presented in the discussion and abstract, without it being too onerous or time-consuming a task. The editorial team should routinely check for reporting bias of all kinds to prevent errors that result in retractions. Hopefully our technique can assist.

## Data Availability

All data generated or analyzed for this study are included in this published article.

## Declarations

### Abbreviations

No abbreviations in text. Abbreviations for Table 1 in Legend.

## References

[1] La Rosa G, Vernooij R, Qureshi M, Polosa R, O’Leary R (2023). Clinical testing of the cardiovascular effects of e-cigarette substitution for smoking: a living systematic review. Intern Emerg Med.

[2] Catalogue of Bias Collaboration. Catalogue of Bias. Oxford: Centre for Evidence Based Medicine. ; 2022. Available from: https://catalogofbias.org/.

[3] Catalogue of Bias Collaboration, Mahtani KR, Chalmers I, Nunan D. Spin Bias. In: Catalogue of Bias. 2019. Available from: https://catalogofbias.org/biases/spin-bias/. Accessed 27 July 2022.

[4] Bradley SH, DeVito NJ, Lloyd KE, Logullo P, Butler JE (2022). Improving medical research in the United Kingdom. BMC Res Notes.

[5] Chiu K, Grundy Q, Bero L (2017). Spin’ in published biomedical literature: a methodological systematic review. PLoS Biol.

[6] Héroux ME, Butler AA, Cashin AG, McCaughey EJ, Affleck AJ, Green MA (2022). Quality output Checklist and Content Assessment (QuOCCA): a new tool for assessing research quality and reproducibility. BMJ Open.

[7] Klein DE, Hawkins B, Schwartz R (2022). Understanding experts’ conflicting perspectives on tobacco harm reduction and e-cigarettes: an interpretive policy analysis. SSM - Qualitative Research in Health.

[8] Balfour DJK, Benowitz NL, Colby SM, Hatsukami DK, Lando HA, Leischow SJ (2021). Balancing consideration of the risks and benefits of e-cigarettes. Am J Public Health.

[9] Carroll DM, Denlinger-Apte RL, Dermody SS, King JL, Mercincavage M, Pacek LR (2021). Polarization within the field of tobacco and nicotine science and its potential impact on trainees. Nicotine Tob Res.

[10] Boutron I, Ravaud P (2018). Misrepresentation and distortion of research in biomedical literature. Proc Natl Acad Sci USA.

[11] Meerpohl JJ, Schell LK, Bassler D, Gallus S, Kleijnen J, Kulig M (2015). Evidence-informed recommendations to reduce dissemination bias in clinical research: conclusions from the OPEN (overcome failure to publish nEgative fiNdings) project based on an international consensus meeting. BMJ open.

[12] Puljak L, Riva N, Parmelli E, González-Lorenzo M, Moja L, Pieper D (2020). Data extraction methods: an analysis of internal reporting discrepancies in single manuscripts and practical advice. J Clin Epidemiol.

[13] Biondi-Zoccai G, Sciarretta S, Bullen C, Nocella C, Violi F, Loffredo L (2019). Acute effects of heat-not-burn, electronic vaping, and traditional tobacco combustion cigarettes: the Sapienza University of Rome-Vascular Assessment of Proatherosclerotic Effects of Smoking (SUR - VAPES) 2 randomized trial. J Am Heart Assoc.

[14] Chaumont M, van de Borne P, Bernard A, Van Muylem A, Deprez G, Ullmo J (2019). Fourth generation e-cigarette vaping induces transient lung inflammation and gas exchange disturbances: results from two randomized clinical trials. Am J Physiol Lung Cell Mol Physiol.

[15] Cravo AS, Bush J, Sharma G, Savioz R, Martin C, Craige S (2016). A randomised, parallel group study to evaluate the safety profile of an electronic vapour product over 12 weeks. Regul Toxicol Pharmacol.

[16] Franzen KF, Willig J, Cayo Talavera S, Meusel M, Sayk F, Reppel M (2018). E-cigarettes and cigarettes worsen peripheral and central hemodynamics as well as arterial stiffness: a randomized, double-blinded pilot study. Vasc Med.

[17] George J, Hussain M, Vadiveloo T, Ireland S, Hopkinson P, Struthers AD (2019). Cardiovascular effects of switching from tobacco cigarettes to electronic cigarettes. J Am Coll Cardiol.

[18] Kerr DMI, Brooksbank KJM, Taylor RG, Pinel K, Rios FJ, Touyz RM (2019). Acute effects of electronic and tobacco cigarettes on vascular and respiratory function in healthy volunteers: a cross-over study. J Hypertens.

[19] Sumartiningsih S, Lin HF, Lin JC (2019). Cigarette smoking blunts exercise-induced heart rate response among young adult male smokers. Int J Environ Res Public Health.

[20] Shaqman M, Al-Abedalla K, Wagner J, Swede H, Gunsolley JC, Ioannidou E (2020). Reporting quality and spin in abstracts of randomized clinical trials of periodontal therapy and cardiovascular disease outcomes. PLoS ONE.

[21] Corcoran A, Neale M, Arthur W, Ottwell R, Roberts W, Hartwell M (2022). Evaluating spin in the abstracts of systematic reviews and meta-analyses on cannabis use disorder. Subst Abus.

[22] Li G, Abbade LPF, Nwosu I, Jin Y, Leenus A, Maaz M (2017). A scoping review of comparisons between abstracts and full reports in primary biomedical research. BMC Med Res Methodol.

[23] Boutron I (2020). Spin in scientific publications: a frequent detrimental research practice. Ann Emerg Med.

[24] Tosatto D, Bonacina D, Signori A, Pellicciari L, Cecchi F, Cornaggia CM (2022). Spin of information and inconsistency between abstract and full text in RCTs investigating upper limb rehabilitation after stroke: an overview study. Restor Neurol Neurosci.

[25] Nascimento DP, Costa LO, Gonzalez GZ, Maher CG, Moseley AM (2019). Abstracts of low back pain trials are poorly reported, contain spin of information, and are inconsistent with the full text: an overview study. Arch Phys Med Rehabil.

[26] Balcerak G, Shepard S, Ottwell R, Arthur W, Hartwell M, Beaman J (2021). Evaluation of spin in the abstracts of systematic reviews and meta-analyses of studies on opioid use disorder. Subst Abus.

[27] Yavchitz A, Ravaud P, Altman DG, Moher D, Hrobjartsson A, Lasserson T (2016). A new classification of spin in systematic reviews and meta-analyses was developed and ranked according to the severity. J Clin Epidemiol.

[28] Gates M, Gates A, Pieper D, Fernandez RM, Tricco AC, Moher D (2022). Reporting guideline for overviews of reviews of healthcare interventions: development of the PRIOR statement. BMJ.

[29] Lancee M, Lemmens CMC, Kahn RS, Vinkers CH, Luykx JJ (2017). Outcome reporting bias in randomized-controlled trials investigating antipsychotic drugs. Transl Psychiatry.

