# Correction: Identifying spin bias of nonsignificant findings in biomedical studies

**DOI:** 10.1186/s13104-023-06441-9

**Published:** 2023-08-24

**Authors:** Renée O’Leary, Giusy Rita Maria La Rosa, Robin Vernooij, Riccardo Polosa

**Affiliations:** 1 Center of Excellence for the Acceleration of Harm Reduction, University of Catania, Via Santa Sofia, 89 Torre Biologica 11 piano, 95123 Catania, Italy; 2 Department of Clinical and Experimental Medicine, University of Catania, Catania, Italy; 3 Department of Nephrology and Hypertension, University Medical Center Utrecht, Utrecht, The Netherlands; 4 Julius Center for Health Sciences and Primary Care, University Medical Center Utrecht, Utrecht University, Utrecht, The Netherlands

BMC Research Notes (2023) 16:50

10.1186/s13104-023-06321-2

The authors have asked to declare the following potential conflict of interest in this published article [1]:

RO did not receive any funding for this study. Within the past five years RO was supported by a contract with ECLAT, Srl, and ECLAT has received funding from the Foundation for a Smoke-Free World.

RP is full tenured professor of Internal Medicine at the University of Catania (Italy) and Medical Director of the Institute for Internal Medicine and Clinical Immunology at the same University. He has received grants from U-BIOPRED and AIR-PROM, Integral Rheumatology & Immunology Specialists Network (IRIS), Foundation for a Smoke Free World, Pfizer, GlaxoSmithKline, CV Therapeutics, NeuroSearch A/S, Sandoz, Merk Sharp & Dohme, Boehringer Ingelheim, Novartis, Arbi Group Srl., Duska Therapeutics, Forest Laboratories and Ministero dell’ Università e della Ricerca (MUR) Bando PNRR 3277/2021 (CUP E63C22000900006) and 341/2022 (CUP E63C22002080006), funded by NextGenerationEU, the European Union (EU) economic recovery package. He is founder of the Center for Tobacco Prevention and Treatment (CPCT) at the University of Catania and of the Center of Excellence for the Acceleration of Harm Reduction at the same university. He receives consultancy fees from Pfizer, Boehringer Ingelheim, Duska Therapeutics, Forest Laboratories, CV Therapeutics, and Sermo Inc. He is being paid textbook royalties from Elsevier. He is also involved in a patent application for ECLAT Srl. He is a pro bono scientific advisor for Lega Italiana Anti Fumo (LIAF) and the International Network of Nicotine Consumers Organizations (INNCO); and he is Chair of the European Technical Committee for Standardization on “Requirements and test methods for emissions of electronic cigarettes” (CEN/TC 437; WG4).

The original article has been corrected.

## References

[1] O’Leary R, et al. Identifying spin bias of nonsignificant findings in biomedical studies. BMC Res Notes. 2023;16:50. 10.1186/s13104-023-06321-2. 10.1186/s13104-023-06321-2 PMC10155298 37131244

